# PReS-FINAL-2169: Exposure-response modeling of canakinumab in the avoidance of flares in children with systemic juvenile idiopathic arthritis

**DOI:** 10.1186/1546-0096-11-S2-P181

**Published:** 2013-12-05

**Authors:** Y Xiong, W Wang, W Ebling, H Sun, O Luttringer, K Abrams, N Ruperto, H Brunner

**Affiliations:** 1 Novartis Pharmaceuticals Corporation, East Hanover, NJ, USA; 2 Novartis Pharma AG, Basel, Switzerland; 3 Istituto Gaslini, Genova, Italy; 4 Children's Hospital Cincinnati, Cincinnati, OH, USA

## Introduction

Canakinumab (CAN), a fully human selective anti-IL-1β monoclonal antibody, has been shown to be efficacious in systemic juvenile idiopathic arthritis (SJIA), resulting in significantly longer times to flare vs. Placebo (PBO).

## Objectives

1) To explore the relationship between SJIA flare reduction and CAN exposure (4 mg/kg/every 4 weeks) with consideration of patient baseline characteristics using a discrete hazard (flare) simulation model. 2) To predict the effects of body weight-tiered CAN dosing regimens at 1 to 6 mg/kg every 4 weeks on SJIA flare rates compared with PBO.

## Methods

Plasma concentrations were modeled for patients treated with CAN (n = 50) or PBO after CAN treatment (n = 50) and used to predict flare risk by a validated and qualified simulation of the CAN exposure-flare hazard relationship. The model considered both PBO and CAN treatments and multiple covariates, including baseline steroid dose, heterogeneity of the population with respect to disease severity (which had a varying influence on risk of an early flare), and declining CAN concentrations over time due to washout in patients on PBO (after receiving CAN). The final simulation model was also used to explore the dose-response relationship between SJIA flare hazard and CAN dose in a simulated trial (1000 simulations), that modeled 700 patients randomized to 1 of 7 treatment arms: PBO, 1 mg/kg, 2 mg/kg, 3 mg/kg, 4 mg/kg, 5 mg/kg, and 6 mg/kg (all every 4 weeks) of CAN.

## Results

The final simulation model successfully re-produced the Kaplan-Meier curves observed in the phase III program, with significant differences in flare hazard (p < 0.001) between treatment arms. Higher CAN plasma concentrations were associated with lower flare hazard. Differences in the corticosteroid dose at baseline, age, gender, body weight, daily steroid usage, and level of adapted ACR response to CAN were not significant predictors of flare risk. Based on simulation, the probability of flare (90% CI) over 12 months was 63% (55%, 71%) for the PBO arm. CAN at 4 mg/kg/every 4 weeks reduced the flare rate over PBO by 39% (28%, 49%), consistent with the clinical data observed. Based on simulation, CAN at 1, 2, 3, 4, 5, and 6 mg/kg every 4 weeks was associated with annual flare rates of 37% (28%, 47%), 30% (24%, 38%), 26% (21%, 33%), 24% (19%, 30%), 22% (17%, 27%), and 21% (16%, 26%), respectively. Relative to the approved CAN dose, the model predicted a change in flare probability of +13%, +6%, +2%, -2%, and -3% for the 1, 2, 3, 5, and 6 mg/kg every 4 weeks doses, respectively.

## Conclusion

The simulations support 4 mg/kg every 4 weeks as the appropriate dose for preventing SJIA flare events. Doses greater than 4 mg/kg provide only marginal gain in flare reduction over 12 months, while doses less than 4 mg/kg relatively increase the risk of experiencing a flare.

## Disclosure of interest

Y. Xiong Employee of: Novartis Pharmaceuticals Corporation, W. Wang Shareholder of: Novartis Pharmaceuticals Corporation, Employee of: Novartis Pharmaceuticals Corporation, W. Ebling Consultant for: Novartis Pharmaceuticals Corporation, H. Sun Shareholder of: Novartis Pharmaceuticals Corporation, Employee of: Novartis Pharmaceuticals Corporation, O. Luttringer Shareholder of: Novartis Pharma AG, Employee of: Novartis Pharma AG, K. Abrams Shareholder of: Novartis Pharmaceuticals Corporation, Employee of: Novartis Pharmaceuticals Corporation, N. Ruperto Grant/Research Support from: Abbot, Astra Zeneca, Bristol Myers and Squibb, Centocor research & development, Eli Lilly and company,"Francesco Angelini" s.p.a, Glaxosmith & Kline, Italframaco, Merck Serono, Novartis, Pfizer, Regeneron, Roche, Sanofi Aventis, Schwarz Biosciences gmbh, Xoma, Wyeth, Speakers Bureau: Abbott, Bristol Myers Squibb, Astellas, Boehringer, Italfarmaco, medimmune, Novartis, Pfizer, Roche, H. Brunner Consultant for: Novartis, Genentech, medimmune, EMD, Serono, AMS, Pfizer, UCB, Jannsen, Speakers Bureau: Genentech.

